# Circunferência do Pescoço e Risco Cardiovascular em 10 Anos na Linha de Base do ELSA-Brasil: Diferenciais por Sexo

**DOI:** 10.36660/abc.20190289

**Published:** 2020-11-01

**Authors:** Acácia Antônia Gomes de Oliveira Silva, Larissa Fortunato de Araujo, Maria de Fátima Haueisen Sander Diniz, Paulo Andrade Lotufo, Isabela Martins Bensenor, Sandhi Maria Barreto, Luana Giatti

**Affiliations:** 1 Universidade Federal de Ouro Preto Campus Morro do Cruzeiro Ouro PretoMG Brasil Universidade Federal de Ouro Preto - Campus Morro do Cruzeiro - Programa de Pós-Graduação em Saúde e Nutrição, Ouro Preto, MG - Brasil; 2 Universidade Federal do Ceará FortalezaCE Brasil Universidade Federal do Ceará, Fortaleza, CE - Brasil; 3 Universidade Federal de Minas Gerais Belo HorizonteMG Brasil Universidade Federal de Minas Gerais, Belo Horizonte, MG - Brasil; 4 Universidade de São Paulo São PauloSP Brasil Universidade de São Paulo, São Paulo, SP – Brasil

**Keywords:** Doenças Cardiovasculares, Fatores de Risco, Gênero, Adiposidade, Risco Cardiovascular

## Abstract

**Fundamento:**

A circunferência do pescoço (CP) é uma medida indireta do tecido adiposo subcutâneo da parte superior do corpo, apontada como um preditor independente de doenças cardiometabólicas.

**Objetivos:**

Verificar a associação entre a CP e o risco cardiovascular em 10 anos (risco de doença cardiovascular [DCV] em 10 anos) em homens e mulheres separadamente.

**Métodos:**

Análise seccional com inclusão de 13.920 participantes da linha de base do Estudo Longitudinal da Saúde do Adulto (ELSA-Brasil). A associação entre a CP (utilizada como variável contínua e agregada em quartis) e o risco de DCV em 10 anos, estimado pelo Framingham Global Risk Score (FGRS), foi investigada por meio de modelos lineares generalizados após ajustes por características sociodemográficas, comportamentos em saúde, índice de massa corporal e circunferência da cintura. O nível de significância estatístico adotado foi de 5%.

**Resultados:**

A média da CP foi de 39,5 cm (desvio-padrão [DP] de ± 3,6) nos homens e 34,0 cm (DP de ±2,9) nas mulheres. Após ajustes, o aumento de 1 cm na CP foi associado ao incremento de 3% (IC 95%: 1,02 a 1,03) e 5% (IC 95%: 1,04 a 1,06) na média aritmética do risco de DCV em homens e mulheres, respectivamente. No último quartil da CP, homens e mulheres apresentaram um incremento de 18% (IC 95%: 1,13 a 1,24) e 35% (IC 95%: 1,28 a 1,43), respectivamente, na média aritmética do risco de DCV após ajustes.

**Conclusões:**

Verificamos associação positiva e independente entre a CP e o risco de DCV em 10 anos. Resultados sugerem que a CP pode contribuir para a predição de risco cardiovascular além daquele observado pelas medidas antropométricas clássicas.

## Introdução

Evidências apontam que a localização do tecido adiposo importa na determinação do risco à saúde.^1^ Sabe-se que a adiposidade localizada na parte superior do corpo está mais fortemente associada a DCV, resistência à insulina e diabetes tipo 2 do que a adiposidade localizada na parte inferior.^2^ Adicionalmente, evidências indicam que a gordura visceral abdominal está ligada, independentemente de outras medidas de adiposidade, ao risco cardiometabólico aumentado,^3^ sendo tal relação mais forte do que a observada com a gordura subcutânea abdominal.^4^

Entretanto, a presença do tecido adiposo visceral abdominal não explica toda a variação nos modelos de risco cardiometabólico, o que sugere que depósitos de gordura em outras localidades possam ser relevantes.^5^ Desse modo, tem crescido o interesse no estudo do risco metabólico associado à gordura subcutânea da parte superior do corpo, especificamente da região do pescoço.^6^

A CP, uma medida antropométrica simples e prática, é considerada um indicador indireto do acúmulo de tecido adiposo subcutâneo na parte superior do corpo.^7^ Sugere-se que a CP represente um risco cardiometabólico adicional, independente de outras medidas de adiposidade.^5^ Resultados de análises seccionais mostraram que ela esteve positivamente associada a síndrome metabólica,^8^ hiperinsulinemia,^9^ pressão arterial elevada^10^ e um conjunto de fatores de risco cardiometabólicos,^11^ após considerar a adiposidade corporal global e a abdominal. Resultados da linha de base do ELSA-Brasil também confirmaram associação positiva entre a CP e fatores de risco cardiometabólicos.^12^ Por essa razão, uma CP aumentada tem sido considerada um fator de risco cardiovascular^13^ e proposta como medida adicional não invasiva de predição desse risco.^14^

O FGRS tem por objetivo predizer o risco de DCV em 10 anos,^15^ sendo utilizado para identificar indivíduos sob maior risco de DCV e orientar a prática clínica.^16^ Considerando que a CP já se mostrou mais fortemente associada à síndrome metabólica em homens e mais fortemente ligada à hipertensão arterial em mulheres,^17^ este estudo investigou se a CP está relacionada ao risco de DCV em 10 anos, estimado pelo FGRS, em homens e mulheres separadamente. Assim, foi investigada a hipótese de que, quanto maior a CP, maior o risco de DCV em 10 anos, e que essa associação é independente do índice de massa corporal (IMC) e da circunferência da cintura, com magnitude diferente entre homens e mulheres.

## Métodos

Foi feita uma análise seccional utilizando dados da linha de base do ELSA-Brasil. Trata-se de uma coorte multicêntrica constituída por servidores públicos ativos e aposentados, de 35 a 74 anos de idade, de instituições de ensino e pesquisa localizadas em seis capitais brasileiras (Belo Horizonte, Porto Alegre, Rio de Janeiro, Salvador, São Paulo e Vitória), que visa investigar os fatores associados ao desenvolvimento e à progressão das DCV e do diabetes. A coorte incluiu voluntários (76% do total da amostra) e participantes recrutados ativamente (24%). Esforços foram feitos para selecionar proporções similares de homens e mulheres e proporções pré-definidas de grupos etários e categorias ocupacionais. Os critérios de exclusão foram a intenção de sair da instituição, mulheres grávidas ou ter estado grávida há menos de quatro meses, apresentar grave dificuldade cognitiva ou de comunicação e, se aposentado, residir fora da região metropolitana correspondente.^18^ Na linha de base (2008-2010), foram incluídos 15.105 participantes, dos quais 54,4% eram mulheres, 52,2% declaravam ter raça/cor de pele branca, e 52,7% tinham grau universitário.^19^ Detalhes sobre o desenho do estudo e o perfil da coorte podem ser encontrados em publicações anteriores.^18^^,^^19^

Os dados foram obtidos por meio de entrevista face a face, aferição de medidas e exames realizados por profissionais treinados e certificados, utilizando instrumentos e procedimentos padronizados. O ELSA-Brasil foi aprovado pelos Comitês de Ética em Pesquisa das instituições de ensino e pesquisa participantes, e todos os participantes assinaram o termo de consentimento livre e esclarecido.

Para o propósito deste estudo, foram excluídos indivíduos com relato de DCV (infarto agudo do miocárdio, insuficiência cardíaca, acidente vascular cerebral e cirurgia cardíaca de revascularização; n = 738) e informações faltantes para DCV (n = 26), para a CP (n = 11), para o FGRS (n = 28) e para covariáveis (n = 382). Ao final, a amostra analítica incluiu 13.920 indivíduos.

### Variável Resposta

A variável resposta foi o risco de DCV em 10 anos (contínua) mensurado pelo FGRS, que estima o risco de desenvolver, em 10 anos, pelo menos um dos seguintes eventos: doença arterial coronariana, eventos cerebrovasculares, doença arterial periférica e insuficiência cardíaca.^15^ O escore é sexo-específico e composto por: idade (em anos), tabagismo atual, colesterol total sérico, HDL-colesterol, pressão arterial sistólica, presença de diabetes e uso de anti-hipertensivos.^15^

Indivíduos que relataram terem fumado pelo menos 100 cigarros ou cinco maços de cigarro na vida, e que ainda fumavam, foram considerados fumantes; os demais foram classificados como não fumantes. A pressão arterial foi mensurada com o dispositivo automático Omron®, usando procedimentos padrões, sendo utilizada a média da segunda e terceira medidas.^20^ Presença de diabetes foi definida pelo autorrelato de diagnóstico médico da doença ou de uso de medicamento para o tratamento dela nas duas últimas semanas, ou níveis de glicemia em jejum ≥ 7,0 mmol/l, ou glicemia após 2 horas de ingestão de solução padronizada de glicose ≥ 11,1 mmol/l, ou hemoglobina glicada (HbA1c) ≥ 6,5%. A glicemia foi mensurada pelo método da hexoquinase (ADVIA Chemistry; Siemens, Deerfield, Illinois), e a HbA1c foi mensurada por cromatografia líquida (Bio-Rad Laboratories, Hercules, Califórnia). O uso de medicamentos anti-hipertensivos e antidiabéticos foi definido com o autorrelato e a verificação de prescrições e embalagens de medicamentos. O colesterol total e o HDL-colesterol foram obtidos utilizando ADVIA 1200 Siemens®. Os parâmetros laboratoriais foram dosados em amostras de sangue após jejum médio de 12 horas (mínimo de 10 e máximo de 14 horas), processadas em um único laboratório.^21^

### Variável Explicativa

A CP foi medida com uma fita inelástica (mm) posicionada acima da cartilagem cricoide e perpendicular ao eixo longo do pescoço, com o participante em posição sentada. Foi utilizada como variável contínua em centímetros e categorizada em quartis.

### Covariáveis

As covariáveis incluíram as características sociodemográficas: idade em faixa etária (35 a 44, 45 a 54, 55 a 64, 65 a 74) na descrição da população e como variável contínua nos modelos de regressão; raça/cor da pele autodeclarada (branca, parda, preta, amarela e indígena) e escolaridade (superior completo, médio completo, fundamental completo e fundamental incompleto).

Incluíram-se também comportamentos relacionados à saúde. O consumo semanal de álcool (“consumo moderado”, “não consome” e “consumo excessivo”) foi obtido por autorrelato do número de doses e tipo de bebida consumidos por semana, transformados em gramas. O consumo excessivo foi definido como ≥ 210 g para homens e ≥ 140 g para mulheres; qualquer consumo abaixo desses valores foi considerado como moderado. A atividade física no lazer foi obtida usando a seção de tempo no lazer da versão longa do *International Physical Activity Questionnaire* (IPAQ), categorizado a partir da soma do tempo em cada tipo de atividade ponderando pela intensidade da mesma (forte: ≥ 3.000 MET-min/semana; moderada: 600 a 3.000 MET-min/semana; fraca: < 600 MET-min/semana).^22^

Outras medidas antropométricas consideradas na análise foram o IMC (kg/m^2^) e a circunferência da cintura em centímetros. Foram descritas como variáveis categóricas, sendo o IMC categorizado em “sem excesso de peso” (IMC < 25), “sobrepeso” (IMC ≥ 25 e < 30) e “obesidade” (IMC ≥ 30). A circunferência da cintura foi categorizada em “adequada” e “inadequada” (≥ 88 cm para as mulheres e ≥ 102 cm para os homens).^23^ As duas medidas foram utilizadas como variáveis contínuas nos modelos de regressão. Essas aferições foram realizadas em jejum, utilizando procedimentos padronizados. A circunferência da cintura foi medida no ponto médio entre a borda inferior do arco costal e a crista ilíaca na linha axilar média.^24^

### Análise de Dados

As características da população do estudo e dos componentes do FGRS foram descritas por meio de frequências absolutas e relativas (variáveis categóricas) e médias e DP ou medianas (1º e 3º quartis), que são variáveis contínuas com e sem distribuição normal, respectivamente. O teste de Shapiro-Wilk foi utilizado para verificar a normalidade das variáveis. Foram utilizados teste de Qui-quadrado de Pearson para comparação de frequências, teste t de *Student* não pareado para comparação de médias e teste de Mann-Whitney para comparação de medianas. Realizou-se análise de variância (ANOVA - Oneway) com teste *pos-hoc* de Bonferroni para estimar diferenças nas médias da CP segundo as categorias de risco de DCV em 10 anos (baixo: < 6%; intermediário: ≥ 6% e ≤ 20%; e alto: > 20%). O nível de significância estatística adotado foi de 5%.

A magnitude da associação entre a CP e o risco de DCV em 10 anos foi estimada por meio de modelos lineares generalizados (MLG), que são uma generalização do modelo linear clássico que permite erros não normais e função de ligação não identificada.^25^ Utilizou-se o MLG com distribuição gama e função de ligação logarítmica. Os resultados representam a razão de média aritmética (RMA) obtida pela exponenciação dos coeficientes da regressão.

Inicialmente, foi estimada a associação bruta entre a CP (contínua) e o risco de DCV em 10 anos (modelo 0). Em seguida, foram estimados modelos multivariados, realizando ajustes sucessivos por: idade, raça/cor da pele e escolaridade atual (modelo 1); atividade física e consumo de bebidas alcoólicas (modelo 2); IMC (modelo 3) e, por fim, circunferência da cintura (modelo 4). Adicionalmente, a magnitude da associação entre a CP e o risco DVC em 10 anos foi estimada utilizando a CP categorizada em quartis, realizando análise multivariada com a mesma sequência de ajustes. Todas as análises foram estratificadas por sexo.

Análises de sensibilidade foram realizadas com a exclusão de participantes em uso de hipolipemiantes e corticoide, bem como de mulheres em uso de anticoncepcional ou reposição hormonal. O uso de medicamentos foi obtido com base em autorrelato e verificação de prescrições médicas e embalagens no dia da entrevista. As análises foram realizadas no *Software Stata* versão 13 (Stata Corporation, College Station, EUA).

## Resultados

Entre 13.920 participantes, 55% eram mulheres, e a idade média foi 51,7 anos (DP ± 7,6). A maioria dos homens e mulheres relatou raça/cor branca e nível superior de escolaridade. A prevalência de sobrepeso foi mais elevada em homens, e a de obesidade, em mulheres. A circunferência da cintura inadequada foi mais frequente nas mulheres do que nos homens (44,3% *versus* 25,5%). A média da CP foi de 39,5 cm (DP ± 3,6) nos homens e 34 cm (DP ± 2,9) nas mulheres (Tabela 1).

**Tabela 1:** Descrição das características da população de estudo em homens e mulheres segundo o ELSA-Brasil, 2008-2010

Características		Homens		Mulheres		Valor de p
		(n = 6.261)		(n = 7.659)		
		n	%	n	%	
**Idade (anos)**						
	35-44	1.481	23,7	1.708	22,3	0,009*
	45-54	2.518	40,2	3.086	40,3	
	55-64	1.634	26,1	2.165	28,3	
	65-75	628	10,0	700	9,1	
**Raça/cor de pele autorreferida**						
	Branca	3.299	52,7	3.981	52	< 0,001*
	Parda	1.888	30,2	2.041	26,7	
	Preta	870	13,9	1.351	17,6	
	Amarela	120	1,9	225	2,9	
	Indígena	84	1,3	61	0,8	
**Escolaridade**						
	Superior completo	3.162	50,5	4.245	55,4	< 0,001*
	Ensino médio completo	2.094	33,5	2.744	35,8	
	Ensino fundamental completo	516	8,2	396	5,2	
	Ensino fundamental incompleto	489	7,8	274	3,6	
**Consumo de álcool**						
	Moderado	3.994	63,8	4.673	61,0	< 0,001*
	Não consome ou é ex-usuário	1.486	23,7	2.718	35,5	
	Excessivo	781	12,5	268	3,5	
**Atividade física no lazer**						
	Leve	4.596	73,4	6.100	79,7	< 0,001*
	Moderado	1086	17,4	1.143	14,9	
	Forte	579	9,2	416	5,4	
**Índice de massa corporal (IMC) (Kg/m^2^**)						
	Sem excesso de peso	2.179	34,8	3.040	39,7	< 0,001*
	Sobrepeso	2.819	45,0	2.756	36,0	
	Obesidade	1.263	20,2	1.863	24,3	
**Circunferência da cintura (CC) (cm)**						
	Adequado	4.662	74,5	4.270	55,7	< 0,001*
	Inadequado	1.599	25,5	3.389	44,3	
	**Circunferência do pescoço (CP) (cm), média (± DP)**	39,5	(±3,6)	34,0	(±2,9)	< 0,001**
	**Escore de risco cardiovascular em 10 anos (%), mediana (1º/3º quartil)**	11,3	(6,2- 19,9)	4,4	(2,4-8,3)	< 0,001***

IMC: sem excesso de peso, < 24,9 kg/m²; sobrepeso, 25,0 a 29,9 kg/m²; obesidade, ≥ 30 kg/m². CC: inadequado, ≥ 88 cm para as mulheres e ≥ 102 cm para os homens; adequado, < 88 cm para as mulheres e < 102 cm para homens.

* Teste Qui-quadrado de Pearson;

** Teste t de Student não pareado;

*** Teste de Mann-Whitney.

**Figura 1:**
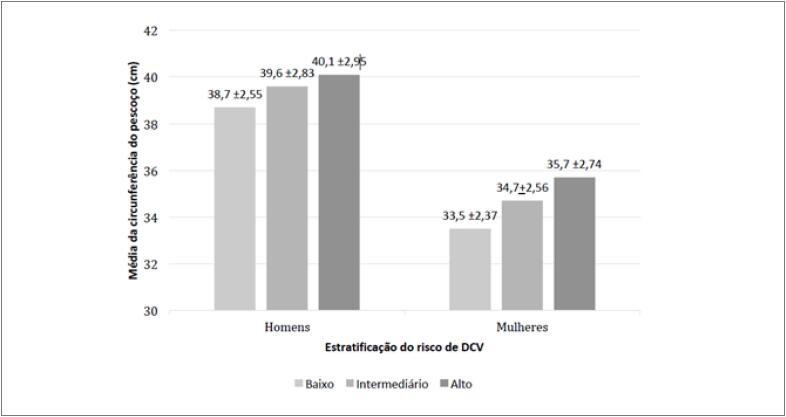
Média (cm) da circunferência do pescoço de acordo com a estratificação do risco de DCV em 10 anos em homens e mulheres, ELSA-Brasil, 2008-2010. Risco baixo: < 6%; risco intermediário: ≥ 6% e ≤ 20%; risco alto: > 20%. Teste ANOVA oneway e teste post hoc de Bonferroni.

A descrição dos componentes do FGRS está apresentada na Tabela 2. A média da CP aumentou com o incremento do risco de DCV em 10 anos agrupado em categorias de risco (baixo: < 6%; intermediário: ≥ 6% e ≤ 20%; alto > 20%) em ambos os sexos (Figura 1).

**Tabela 2:** Descrição dos componentes do Framingham Global Risk Score em homens e mulheres segundo o ELSA-Brasil, 2008-2010

Fatores de risco	Homens (n = 6.261)		Mulheres (n = 7.659)		Valor de p
Idade (anos), média (± DP)	51,7	± 11,5	51,8	± 10,0	0,53*
Colesterol total (mg/dl), mediana (1º e 3º quartis)	210	(185-239)	214	(189-241)	< 0,001**
Colesterol HDL (mg/dl), mediana (1º e 3º quartis)	49	(43-57)	60	(51-70)	< 0,001**
Uso de anti-hipertensivo, n (%)	1.687	26,9	2.071	27,0	0,90***
Pressão arterial sistêmica, média (± DP)	125,3	± 20,9	117,2	± 18,9	< 0,001*
Presença de diabetes, n (%)	1.200	19,2	1.068	14,0	< 0,001***
Presença de tabagismo, n (%)	889	14,4	927	12,1	< 0,001***

* Teste t de Student não pareado;

** Teste de Mann-Whitney;

*** Teste Qui-quadrado de Pearson.

Os resultados dos modelos de regressão com a variável CP contínua são apresentados na Tabela 3. Em homens, observou-se que o aumento de 1 cm na CP foi associado ao incremento de 5% (IC 95%: 1,04 a 1,05) na média aritmética do risco de DCV em 10 anos (modelo 0). Essa associação se manteve estatisticamente significativa após todos os ajustes (RMA: 1,03; IC 95%: 1,02 a 1,03) (modelo 4). Já entre as mulheres, o aumento de 1 cm da CP foi associado ao incremento de 11% na média aritmética do risco de DCV em 10 anos (IC 95%: 1,10 a 1,12) (modelo 0). Após todos os ajustes (modelo 4), o incremento de 1 cm da CP foi associado ao aumento de 5% (IC 95%: 1,04 a 1,06) na média aritmética do risco de DCV em 10 anos (Tabela 3).

**Tabela 3:** Análise multivariada entre a circunferência do pescoço e o risco de desenvolver DCV em 10 anos em homens e mulheres, ELSA-Brasil, 2008-2010

	Homens	Mulheres
	RMA (IC 95%)	RMA (IC 95%)
Modelo 0	1,05 (1,04 – 1,05)	1,11 (1,10 – 1,12)
Modelo 1	1,06 (1,05 – 1,06)	1,09 (1,08 – 1,10)
Modelo 2	1,06 (1,05 – 1,06)	1,09 (1,08 – 1,10)
Modelo 3	1,03 (1,02 – 1,04)	1,07 (1,06 – 1,08)
Modelo 4	1,03 (1,02 – 1,03)	1,05 (1,04 – 1,06)

RMA (IC 95%): razão de médias aritméticas (RMA) obtidas por meio dos modelos lineares generalizados e seu intervalo com 95% de confiança. Modelo 0: RMA não ajustada; modelo 1: ajuste por idade, raça/cor autorreferida e escolaridade; modelo 2: modelo 1 + ajuste por consumo de álcool e atividade física no lazer; modelo 3: modelo 2 + ajuste por índice de massa corporal; modelo 4: modelo 3 + circunferência da cintura.

Os resultados dos modelos de regressão utilizando a CP agrupada em quartis são apresentados na Tabela 4. Após todos os ajustes, observou-se que, comparados ao primeiro quartil, todos os demais apresentaram aumento gradual na média aritmética no risco de DCV em 10 anos, que chegou a um incremento de 18% entre os que estavam no último quartil (IC 95%: 1,13 a 1,24) entre os homens e a 35% (IC 95%: 1,28 a 1,43) entre as mulheres (modelo 4).

**Tabela 4:** Análise multivariada entre a circunferência do pescoço agrupada em quartis e o risco de DCV em 10 anos em homens e mulheres, ELSA-Brasil, 2008-2010

	Homens RMA (IC 95%)				Mulheres RMA (IC 95%)			
	Q1	Q2	Q3	Q4	Q1	Q2	Q3	Q4
Modelo 0	1,00	1,14 (1,08-1,21)	1,21 (1,14-1,28)	1,42 (1,34-1,51)	1,00	1,23 (1,16-1,31)	1,59 (1,49-1,70)	2,04 (1,91-2,18)
Modelo 1	1,00	1,14 (1,10-1,19)	1,30 (1,25-1,35)	1,52 (1,47-1,58)	1,00	1,16 (1,11-1,21)	1,37 (1,31-1,43)	1,78 (1,71-1,86)
Modelo 2	1,00	1,14 (1,10-1,18)	1,28 (1,24-1,33)	1,49 (1,43-1,54)	1,00	1,16 (1,11-1,20)	1,36 (1,30-1,41)	1,76 (1,69-1,84)
Modelo 3	1,00	1,07 (1,04-1,11)	1,16 (1,11-1,21)	1,24 (1,18-1,30)	1,00	1,10 (1,06-1,15)	1,25 (1,19-1,31)	1,50 (1,42-1,58)
Modelo 4	1,00	1,05 (1,02-1,09)	1,12 (1,08-1,17)	1,18 (1,13-1,24)	1,00	1,06 (1,02-1,11)	1,17 (1,12-1,23)	1,35 (1,28-1,43)

RMA (IC 95%): razão de médias aritméticas (RMA) obtidas por meio dos modelos lineares generalizados e seu intervalo com 95% de confiança. Q1, Q2, Q3, Q4: intervalo interquartil. Modelo 0: RMA não ajustada; modelo 1: ajuste por idade, raça/cor autorreferida e escolaridade; modelo 2: modelo 1 + ajuste por consumo de álcool e atividade física no lazer; modelo 3: modelo 2 + ajuste por índice de massa corporal; modelo 4: modelo 3 + circunferência da cintura.

As análises de sensibilidade com a exclusão de participantes em uso de hipolipemiantes e corticoides e de mulheres em uso de anticoncepcional ou em reposição hormonal não alteraram os resultados observados.

## Discussão

Os resultados deste estudo apontam uma associação direta entre o aumento da CP e o incremento na estimativa do risco de DCV em 10 anos, independentemente de potenciais fatores de confusão e de outras medidas de adiposidade corporal, especificamente o IMC e a circunferência da cintura, em participantes livres de DCV. Resultados similares foram observados ao se analisar a CP agrupada em quartis, que apresentou a indicação de um gradiente dose-resposta, reforçando a associação observada. A magnitude das associações entre CP (contínua e em quartis) e o risco de DCV em 10 anos foi mais elevada nas mulheres do que nos homens.

Os resultados identificaram associação direta entre a CP e o risco de DCV em 10 anos. Foi encontrado apenas um estudo que investigou a relação entre a CP e a predição de risco de doença arterial coronariana em 10 anos estimado pelo *Framingham Coronary Artery Disease Risk Score*. Esse estudo, que incluiu apenas 100 indivíduos livres de DCV, apontou correlação positiva entre o aumento da CP e do risco de doença arterial coronariana em 10 anos.^14^ Todavia, estudos prévios apontaram associação positiva e independente entre a CP e o espessamento da camada média intimal (IMT),^26^ medida de aterosclerose subclínica preditora de risco cardiovascular. Análise da linha de base do ELSA-Brasil também mostrou associação entre a CP e a IMT, mas não encontrou relação entre CP e calcificação da artéria coronariana, outra medida de aterosclerose subclínica.^27^^,^^28^

Os mecanismos pelos quais o tecido adiposo da região do pescoço pode contribuir para a ocorrência de desfechos cardiovasculares ainda não estão estabelecidos.^29^ O tecido adiposo da região do pescoço é considerado como gordura ectópica,^1^ o que pode explicar parte do seu efeito sistêmico. A formação de depósitos ectópicos de tecido adiposo em vários órgãos, inclusive no tecido adiposo subcutâneo do pescoço, ocorre devido à deposição de triglicérides em células de tecidos não adiposos, que normalmente contém pequenas quantidades de gorduras e parece ser particularmente relevante para o risco cardiovascular,^30^^,^^31^ especialmente a gordura ectópica pericárdica e do fígado.^32^ A atividade disfuncional da gordura ectópica está associada a estresse oxidativo, disfunção endotelial e liberação de citocinas pró-inflamatórias e redução da liberação das adiponectinas anti-inflamatórias, dando início à inflamação crônica e alteração do metabolismo lipídico^33^ envolvido no processo aterosclerótico. Evidências suportam a associação entre a maior CP e os marcadores inflamatórios, notadamente fatores do complemento sérico C-3 e C-4, proteína C reativa, interleucina-6 e fator de necrose tumoral alfa (TNF-α),^34^ e marcadores de disfunção endotelial como a E-seletina.^9^ Ademais, a gordura ectópica parece ser um componente fundamental que diferencia obesos metabolicamente saudáveis de obesos não metabolicamente saudáveis.^26^

Adicionalmente, a maioria dos vasos sanguíneos, incluindo as artérias carótidas, está envolvida pelo tecido adiposo perivascular, que auxilia a regulação do tônus vascular e da função endotelial.^35^ À medida que esse tecido aumenta e se torna disfuncional, há uma ação pró-inflamatória direta nas artérias carótidas, o que poderia explicar o maior risco cardiovascular relacionado ao incremento da CP.^30^ Vale ressaltar ainda que o aumento da CP é um fator de risco reconhecido para a apneia obstrutiva do sono, que, por sua vez, está associado a maior risco de DCV e diabetes tipo 2.^36^

Nossos achados mostraram associações mais fortes entre a CP e o risco de DCV em 10 anos nas mulheres. Corroborando isso, uma análise do *Framingham Heart Study* identificou que a CP elevada estava mais fortemente ligada à dislipidemia e hipertensão nas mulheres,^11^ e maior risco de desenvolver diabetes associado ao aumento da CP foi observado no sexo feminino.^37^ Por outro lado, a CP foi mais fortemente relacionada ao risco de doença arterial coronariana em 10 anos estimado pelo Framingham *Coronary Artery Disease Risk Score* nos homens do que nas mulheres.^14^ Ausência de diferença de gênero nas magnitudes das associações entre CP e alterações cardiometabólicas também foi relatada.^10^

É possível que os padrões de acúmulo de gordura no pescoço,^38^ as diferenças na distribuição da adiposidade subcutânea^39^ e o metabolismo dos ácidos graxos livres^11^ expliquem os diferenciais de sexo observados neste e em outros estudos. Em mulheres, há tendência ao maior acúmulo de tecido adiposo subcutâneo, enquanto nos homens, há maiores depósitos de gordura visceral.^40^ O tecido adiposo subcutâneo na parte superior do corpo libera mais ácidos graxos livres na circulação sistêmica do que o visceral,^41^ e altos níveis deles no plasma contribuem para o aumento da resistência à insulina, da produção de lipoproteínas de muito baixa densidade (VLDL, do inglês *very low density lipoprotein*) e de triglicerídeos, além de induzir o estresse oxidativo e estar associado ao aumento da pressão arterial.^11^^,^^41^ Sabe-se também que o tecido adiposo cervical está distribuído em três compartimentos distintos (tecido adiposo cervical subcutâneo, tecido adiposo cervical posterior e tecido adiposo cervical perivertebral), que parecem influenciar de maneira diferente o risco cardiovascular.^38^ As mulheres têm forte tendência a armazenar tecido adiposo no compartimento subcutâneo na região do pescoço, enquanto homens têm maior armazenamento nos outros locais.^30^ Parece que o tecido adiposo dos compartimentos subcutâneo e cervical posterior está mais fortemente associado ao risco cardiometabólico, especialmente entre as mulheres.^38^

Nossos resultados mostraram que as mulheres apresentaram mediana do escore de risco cardiovascular em 10 anos inferior à dos homens, o que pode ser explicado por níveis de HDL-colesterol mais altos, média de pressão sistólica mais baixa e menor prevalência de diabetes e tabagismo observada nas mulheres. Esses resultados corroboram diferenciais de gênero conhecidos na exposição aos fatores de risco cardiovascular^30^^,^^38^^,^^40^^,^^41^ e ao maior cuidado com a saúde, incluindo maior uso dos serviços de saúde observado em mulheres.^42^

Os resultados apresentados neste estudo apontam que o aumento da CP pode contribuir para a predição do risco de DCV em 10 anos, independentemente do IMC e da circunferência da cintura, medidas de adiposidade mais frequentemente estudadas. Alguns autores apontam vantagens da CP em relação à circunferência da cintura, pois é uma medida mais simples, de mais fácil execução e menos sujeita a erro de medida.^43^ É interessante ressaltar que os achados sugerem que a CP pode estar mais fortemente associada ao risco de DCV em 10 anos. Isso porque, após todos os ajustes, o aumento de 1 cm na CP foi associado a maior incremento na média aritmética do risco de DCV do que o aumento de 1 cm da circunferência da cintura (mulheres: 5% [RMA: 1,05; IC 95%: 1,04 a 1,06] *versus* 3% [RMA: 1,03; IC 95%: 1,01 a 1,02]; homens: 3% [RMA: 1,03; IC 95%: 1,02 a 1,03] *versus* 1% [RMA: 1,01; IC 95%: 1,01 a 1,02], respectivamente). Ressalta-se também que meta-análise recente reforça a associação entre a CP e doença arterial coronariana.^44^

Adicionalmente, o estudo apresenta pontos fortes, como o tamanho da amostra, o rigor metodológico, a possibilidade de ajustes por potenciais fatores de confusão e, por fim, o fato de investigar a associação entre a CP e o risco de DCV em 10 anos, mensurado pelo *FGRS*, um escore reconhecidamente preditor de risco cardiovascular e já incorporado à prática clínica, o qual, como a CP, não exige medidas invasivas.

Limitações a ser pontuadas incluem a natureza seccional da análise, a ausência de validação do *FGRS* para a população brasileira e o fato de a CP ter sido mensurada apenas uma vez. Além disso, não se pode descartar a ocorrência de confusão residual.

## Conclusão

Neste estudo foi identificada uma associação positiva entre o aumento da CP e o risco cardiovascular em 10 anos, independentemente de medidas de adiposidade global e visceral. Esses achados sugerem que a CP pode contribuir para estimar o risco cardiovascular para além das medidas antropométricas clássicas (IMC e circunferência da cintura). Análises longitudinais poderão contribuir com novas evidências acerca do seu papel causal no risco cardiovascular.

## References

[1] 1. Lim S, Meigs JB. Ectopic fat and cardiometabolic and vascular risk. Int J Cardiol. 2013;169(3):166-76. 10.1016/j.ijcard.2013.08.077 24063931

[2] 2. Karpe F, Pinnick KE. Biology of upper-body and lower-body adipose tissue-link to whole-body phenotypes. Nat Rev Endocrinol. 2014;11(2):90-100. 10.1038/nrendo.2014.185 25365922

[3] 3. Lim S, Meigs JB. Links between ectopic fat and vascular disease in humans. Thromb Vasc Biol. 2014;34(9):1820-6. 10.1161/ATVBAHA.114.303035 PMC4140970 25035342

[4] 4. Fox CS, Massaro JM, Hoffmann U, Pou KM, Maurovich-Horvat P, Liu CY, et al. Abdominal visceral and subcutaneous adipose tissue compartments: association with metabolic risk factors in the Framingham Heart Study. Circulation. 2007;116(1):39-48. 10.1161/CIRCULATIONAHA.106.675355 17576866

[5] 5. Lee JJ, Pedley A, Therkelsen KE, Hoffman U, Massaro JM, Levy D, et al. Upper body subcutaneous fat is associated with cardiometabolic risk factors. Am J Med. 2017;130(8):958-66. 10.1016/j.amjmed.2017.01.044 PMC5522762 28238696

[6] 6. Rosenquist KJ, Therkelsen KE, Massaro JM, Hoffman U, Fox CS. Development and reproducibility of a computed tomography-based measurement for upper body subcutaneous neck fat. J Am Heart Assoc. 2014;3(6):e000979. 10.1161/JAHA.114.000979 PMC4338686 25523152

[7] 7. Stabe C, Vasques ACJ, Lima MMO, Tambascia MA, Pareja JC, Yamanaka A, et al. Neck circumference as a simple tool for identifying the metabolic syndrome and insulin resistance: results from the Brazilian Metabolic Syndrome Study. Clin Endocrinol. 2013;78(6):874-81. 10.1111/j.1365-2265.2012.04487.x 22804918

[8] 8. Cizza G, Piaggi P, Lucassen EA, Jonge L, Walter M, Mattingly MS, et al. Obstructive sleep apnea is a predictor of abnormal glucose metabolism in chronically sleep deprived obese adults. PLoS One. 2013;8(5):e65400. 10.1371/journal.pone.0065400 PMC3667085 23734252

[9] 9. Almeida Pititto B, Silva IT, Goulart AC, Fonseca MIH, Bittencourt MS, Santos RD, et al. Neck circumference is associated with non-traditional cardiovascular risk factors in individuals at low-to-moderate cardiovascular risk: cross-sectional analysis of the Brazilian Longitudinal Study of Adult Health (ELSA-Brasil). Diabetol Metab Syndr. 2018 Nov 20;10:82. 10.1186/s13098-018-0388-4 PMC6247673 30479668

[10] 10. Zhou JY, Ge H, Zhu MF, Wang LJ, Chen L, Tan YZ, et al. Neck circumference as an independent predictive contributor to cardio-metabolic syndrome. Cardiovasc Diabetol. 2013 May 16;12:76. 10.1186/1475-2840-12-76 PMC3661343 23680280

[11] 11. Preis SR, Massaro JM, Hoffmann U, D'Agostino Sr RB, Levy D, Robins SJ, et al. Neck circumference as a novel measure of cardiometabolic risk: the Framingham Heart Study. J Clin Endocrinol Metab. 2010;95(8):3701-10. 10.1210/jc.2009-1779 PMC2913042 20484490

[12] 12. Baena CP, Lotufo PA, Fonseca MGM, Santos IS, Goulart AC, Benseñor IMJ. Neck circumference is independently associated with cardiometabolic risk factors: cross-sectional analysis from ELSA-Brasil. Metab Syndr Relat Disord. 2016;14(3):145-53. 10.1089/met.2015.0083 26824404

[13] 13. Bruyndonck L, Vrints CJ. Assessing cardiovascular risk - should physicians start measuring neck circumference? Eur J Prev Cardiol. 2017;24(6):1774-5. 10.1177/2047487317737630 29053014

[14] 14. Koppad AK, Kaulgud RS, Arun BS. A study of correlation of neck circumference with Framingham risk score as a predictor of coronary artery disease. J Clin Diag Res. 2017;11(9):17-20. 10.7860/JCDR/2017/25710.10609 PMC5713775 29207753

[15] 15. D'Agostino RB, Vasan RS, Pencina MJ, Wolf PA, Cobain M, Massaro JM, et al. General cardiovascular risk profile for use in primary care: the Framingham Heart Study. Circulation. 2008;117(6):743-53. 10.1161/CIRCULATIONAHA.107.699579 18212285

[16] 16. D'Agostino RB, Pencina MJ, Massaro JM, Coady S. Cardiovascular disease risk assessment: insights from Framingham. Glob Heart. 2013;8(1):11-23. 10.1016/j.gheart.2013.01.001 PMC3673738 23750335

[17] 17. Yan Q, Sun D, LI X, Zheng Q, Li L, Gu C, et al. Neck circumference is a valuable tool for identifying metabolic syndrome and obesity in Chinese Elder subjects: a community-based study. Diabetes Metab Res Rev. 2014;30(1):69-76. 10.1002/dmrr.2464 23996612

[18] 18. Schmidt MI, Griep RH, Passos VM, Luft VC, Goulart AC, Menezes GMS, et al. Strategies and development of quality assurance and control in the ELSA-Brasil. Rev Saude Publica. 2013;47(Suppl 2):105-12. 10.1590/s0034-8910.2013047003889 24346727

[19] 19. Aquino EML, Barreto SM, Bensenor IM, Carvalho MS, Chor D, Duncan BB, et al. Brazilian Longitudinal Study of Adult Health (ELSA-Brasil): objectives and design. Am J Epidemiol. 2012;175(4):315-24. 10.1093/aje/kwr294 22234482

[20] 20. Mill JG, Pinto K, Griep RH, Goulart A, Foppa M, Lotufo PA, et al. Medical assessments and measurements in ELSA-Brasil. Rev Saude Publica. 2013;47(Suppl 2):54-62. 10.1590/s0034-8910.2013047003851 24346721

[21] 21. Fedeli LG, Vidigal PG, Leite CM, Castilhos CD, Pimentel RA, Maniero VC, et al. Logistics of collection and transportation of biological samples and the organization of the central laboratory in the ELSA-Brasil. Rev Saude Publica. 2013;47(Suppl 2):63-71. 10.1590/s0034-8910.2013047003807 24346722

[22] 22. Guidelines for Data Processing and Analysis of the International Physical Activity Questionnaire (IPAQ) — Short and long forms; 2002.

[23] 23. World Health Organization. Waist circumference and waist-hip ratio: report of a WHO expert consultation. Geneva: WHO; 2008.

[24] 24. Lohman TG, Roche AF, Martorell R. Anthropometric standardization reference manual. Champaign (IL): Human Kinetics Books; 1988.

[25] 25. McCullagh P, Nelder JA. Generalized linear models. 2nd ed. London: Chapman & Hall; 1989.

[26] 26. Rosenquist KJ, Massaro JM, Pencina KM, D'Agostino RB, Beiser A, O'Connor GT, et al. Neck circumference, carotid wall intima-media thickness, and incident stroke. Diabetes Care. 2013;36(9):153-4. 10.2337/dc13-0379 PMC3747939 23970728

[27] 27. Baena CP, Lotufo PA, Santos IS, Goulart AC, Bittencourt MS, Duncan BB, et al. Neck circumference is associated with carotid intimal-media thickness but not with coronary artery calcium: Results from the ELSA-Brasil. Nutr Metab Cardiovasc Dis. 2016;26(3):216-22. 10.1016/j.numecd.2016.01.004 26874907

[28] 28. Santos IS, Alencar AP, Rundek T, Goulart AC, Barreto SM, Pereira AC, et al. Low impact of traditional risk factors on carotid intima-media thickness: the ELSA-Brasil cohort. Arterioscler Thromb Vasc Biol. 2015;35(9):2054-9. 10.1161/ATVBAHA.115.305765 26183615

[29] 29. Aoi S, Miyake T, Iida T, Ikeda H, Ishizaki F, Chikamura C, et al. Association of changes in neck circumference with cardiometabolic risk in postmenopausal healthy women. J Atheroscler Thromb. 2016;23(6):728-36. 10.5551/jat.31963 PMC7399285 26797264

[30] 30. Jaksic VP, Grizelj D, Livun A, Boscic D, Ajduk M, Kusec R, et al. Neck adipose tissue-tying ties in metabolic disorders. Horm Mol Biol Clin Investig. 2018;33(2):1-9. 10.1515/hmbci-2017-0075 29425108

[31] 31. Mittendorfer B. Origins of metabolic complications in obesity: adipose tissue and free fatty acid trafficking. Curr Opin Clin Nutr Metab Care. 2011;14(6):535-41. 10.1097/MCO.0b013e32834ad8b6 PMC3711689 21849896

[32] 32. Morelli M, Gaggini M, Daniele G, Marraccini P, Sicari R, Gastaldelli A. Ectopic fat: the true culprit linking obesity and cardiovascular disease? Thromb Haemost. 2013;110(4):651-60. 10.1160/TH13-04-0285 23884194

[33] 33. Smith U, Hammarstedt A. Antagonistic effects of thiazolidinediones and cytokines in lipotoxicity. Biochim Biophys Acta. 2010;1801(3):377-80. 10.1016/j.bbalip.2009.11.006 19941972

[34] 34. Castro-Pinero J, Delgado-Alfonso A, Gracia-Marco L, Gómez-Martínez S, Esteban-Cornejo I, Veiga OL, et al. Neck circumference and clustered cardiovascular risk factors in children and adolescents: cross-sectional study. BMJ Open. 2017;7(9):e016048. 10.1136/bmjopen-2017-016048 PMC5640146 28899889

[35] 35. Nosalski R, Guzik TJ. Perivascular adipose tissue inflammation in vascular disease. Br J Pharmacol. 2017;174(20):3496-513. 10.1111/bph.13705 PMC5610164 28063251

[36] 36. Kawaguchi Y, Fukumoto S, Inaba M, Koyama H, Shoji T, Shoji S, et al. Different impacts of neck circumference and visceral obesity on the severity of obstructive sleep apnea syndrome. Obesity. 2011;19(2):276-82. 10.1038/oby.2010.170 20706203

[37] 37. Cho NH, Oh TJ, Kim KM, Choi SH, Lee JH, Park KS, et al. Neck circumference and incidence of diabetes mellitus over 10 years in the Korean Genome and Epidemiology Study (KoGES). Sci Rep. 2015 Dec 18;5:18565. 10.1038/srep18565 PMC4683519 26681338

[38] 38. Torriani M, Gill CM, Daley S, Oliveira AL, Azevedo DC, Bredella MA, et al. Compartmental neck fat accumulation and its relation to cardiovascular risk and metabolic syndrome. Am J Clin Nutr. 2014;100(5):1244-51. 10.3945/ajcn.114.088450 PMC6443294 25332322

[39] 39. Liu J, Fox CS, Hickson DA, May WD, Hairstorn KG, Carr JJ, et al. Impact of abdominal visceral and subcutaneous adipose tissue on cardiometabolic risk factors: the Jackson Heart Study. J Clin Endocrinol Metab. 2010;95(12):5419-26. 10.1210/jc.2010-1378 PMC2999970 20843952

[40] 40. Sugiyama MG, Agellon LB. Sex differences in lipid metabolism and metabolic disease risk. Biochem Cell Biol. 2012;90(2):124-41. 10.1139/o11-067 22221155

[41] 41. Nielsen S, Guo Z, Johnson CM, Hensrud DD, Jensen MD. Splanchnic lipolysis in human obesity. J Clin Invest. 2004;113(11):1582-8. 10.1172/JCI21047 PMC419492 15173884

[42] 42. Travassos C, Viacava F, Pinheiro R, Brito A. Utilization of health care services in Brazil: gender, family characteristics, and social status. Rev Panam Salud Publica. 2002;11(5-6):365-73. 10.1590/s1020-49892002000500011 12162833

[43] 43. Ataie-Jafari A, Namazi N, Djalalinia S, Chaghamirzayi P, Abdar ME, Zadehe SS, et al. Neck circumference and its association with cardiometabolic risk factors: a systematic review and meta-analysis. Diabetol Metab Syndr. 2018 Sep 29;10:72. 10.1186/s13098-018-0373-y PMC6162928 30288175

[44] 44. Yang GR, Dye TD, Zand MS, Fogg TT, Yuan SY, Yang JK, et al. Association between neck circumference and coronary heart disease: a meta-analysis. Asian Pac Isl Nurs J. 2019;4(1):34-46. 10.31372/20190401.1031 PMC6484201 31037271

