# Supplementary material for: Circunferência do Pescoço e Risco Cardiovascular em 10 Anos na Linha de Base do ELSA-Brasil: Diferenciais por Sexo
Source: Arq Bras Cardiol. 2020 Nov 1;115(5):840–8. [Article in Portuguese] doi: 10.36660/abc.20190289 (PMC8452203; doi:10.36660/abc.20190289)
Supplement: Translation

# Translation: Neck Circumference and 10-Year Cardiovascular Risk at the Baseline of the ELSA-Brasil Study: Difference by Sex

**Authors:** Acácia Antônia Gomes de Oliveira Silva, Larissa Fortunato de Araujo, Maria de Fátima Haueisen Sander Diniz, Paulo Andrade Lotufo, Isabela Martins Bensenor, Sandhi Maria Barreto, Luana Giatti

**Affiliations:** 1 Ouro PretoMG Brazil Universidade Federal de Ouro Preto - Campus Morro do Cruzeiro - Programa de Pós-Graduação em Saúde e Nutrição, Ouro Preto, MG - Brazil; 2 FortalezaCE Brazil Universidade Federal do Ceará, Fortaleza, CE - Brazil; 3 Belo HorizonteMG Brazil Universidade Federal de Minas Gerais, Belo Horizonte, MG - Brazil; 4 São PauloSP Brazil Universidade de São Paulo, São Paulo, SP – Brazil

**Keywords:** Cardiovascular Diseases, Risk Factors, Gender, Adiposity, Cardiovacular Risk

## Abstract

**Background:**

Neck circumference (NC), an indirect measure of upper-body subcutaneous adipose tissue, has been pointed out as an independent predictor of cardiometabolic diseases.

**Objectives:**

To assess the association between NC and 10-year cardiovascular risk in men and in women.

**Methods:**

Cross-sectional analysis of 13,920 participants of the (baseline) Longitudinal Study of Adult Health (ELSA-Brasil). The association between NC (used as continuous variable and grouped into quartiles) and the 10-year cardiovascular risk was estimated by the Framingham Global Risk Score and analyzed by generalized linear models after adjustments for sociodemographic characteristics, health behaviors, body mass index and waist circumference. The significance level adopted was 5%.

**Results:**

Mean NC was 39.5 cm (SD± 3.6) in men and 34.0 cm (SD±2.9) in women. After adjustments, a one-centimeter increase in NC was associated with an increment of 3% (95%CI1.02-1.03) and 5% (95% 1.04-1.05) in the arithmetic mean of the 10-year CVD risk in men and women, respectively. Men and women in the last quartile showed an increment of 18% (95%CI 1.13-1.24) and 35% (95%CI 1.28-1.43), respectively in the arithmetic mean of the 10-year CVD risk, after adjustments.

**Conclusions:**

We found a positive, independent association between NC and the 10-year cardiovascular disease risk. NC may contribute to the prediction of cardiovascular risk, over and above traditional anthropometric measures.

## Introduction

Evidence has shown that the localization of adipose tissue is important to determine health risk.^1^ It is known that upper-body adiposity is more strongly associated with cardiovascular disease (CVD), insulin resistance and type 2 diabetes as compared with lower-body adiposity.^2^ In addition, independently of other measures of adiposity, abdominal visceral fat seems to be associated with increased cardiometabolic risk,^3^ and this association is stronger than abdominal subcutaneous fat.^4^

However, the presence of abdominal visceral fat does not explain all the variation between cardiometabolic risk models, suggesting that fat deposition in other compartments may be also relevant.^5^ The interest in the study of the metabolic risk associated with upper-body subcutaneous fat, in particular with subcutaneous neck fat, has grown.^6^

Neck circumference (NC), a simple and practical anthropometric measure, is consider an indirect indicator of subcutaneous adipose tissue accumulation in the upper part of the body.^7^ It has been suggested that NC represents an additional cardiometabolic risk, independently of other adiposity measures.^5^ Results of sectional analysis showed that NC was positively associated with metabolic syndrome,^8^ hyperinsulinemia,^9^ elevated blood pressure^10^ and many cardiometabolic risk factors,^11^ after adjustment for abdominal and total body fat. Baseline results of the Brazilian Longitudinal Study of Adult Health (ELSA-Brasil) also confirmed the positive association between NC and cardiometabolic risk factors.^12^ For this reason, an elevated NC has been considered a cardiovascular risk factor^13^ and proposed as an additional non-invasive measure in cardiovascular risk prediction.^14^

The Framingham Global Risk Score (FGRS), designed to predict the 10-year^15^ CVD risk, has been used to identify individuals at greater cardiovascular risk and guide the clinical practice.^16^ Considering that NC has been shown to be more strongly associated with metabolic syndrome in men and with arterial hypertension in women,^17^ the present study investigated whether NC is associated with 10-year CVD risk, estimated by the FGRS, in men and in women separately. We investigated the hypothesis that the higher the NC the greater the 10-year CVD risk, and that this association, with different magnitude to men and women, is independent of body mass index (BMI) and waist circumference (WC)..

## Methods

This was a cross-sectional analysis of baseline data obtained from the ELSA-Brasil. ELSA-Brasil is a multicenter cohort study of active and retired civil servants, aged from 35 to 74 years, from education and research institutions located in six capital cities of Brazil – Belo Horizonte, Porto Alegre, Rio de Janeiro, Salvador, São Paulo and Vitoria. The ELSA-Brasil aims to investigate the factors associated with the development and progression of CVD and diabetes. The cohort was composed of both volunteers (76%) and participants actively recruited (24%). Efforts have been made to recruit similar proportions of men and women, as well as predefined proportions of age groups and occupational categories. Exclusion criteria were willingness to leave the institution, pregnant women, or pregnancy in the last four months, severe cognitive or communication impairment, and retired individuals living outside the metropolitan area.^18^ At baseline (2008-2010), a total of 15,105 participants were included, 54.4% of them were women, 52.2% self-reported as white race, and 52.7% had university education.^19^ Details of design and cohort characteristics of the ELSA-Brasil study can be found in previous publications.^18^^,^^19^

Data were obtained by face-to-face interview, anthropometric measurements and tests performed by trained experts, using standardized instruments and procedures. The ELSA-Brasil was approved by the ethics committees of the institutions involved, and all participants signed and informed consent form.

For the purpose of this study, patients with previous CVD (acute myocardial infarction, heart failure, stroke and cardiac revascularization surgery, n=26), and patients with missing data regarding CVD (n=26), NC (n=11), FGRS (n=28) and covariables (n=382) were excluded. The final analytical sample included 13,920 individuals.

### Response Variable

The (continuous) response variable was the 10-year risk of developing one of the following cardiovascular events – coronary artery disease, cerebrovascular events, peripheral artery disease and heart failure – estimated by the FGRS.^15^ This score is sex-specific and composed of: age (years), current smoking, total serum cholesterol level, HDL-cholesterol, systolic arterial pressure, diabetes and use of anti-hypertensive drugs.^15^

Individuals who ever smoked at least 100 cigarettes (or five packs of cigarettes) and current smokers were classified as “smokers”, and the others as “non-smokers”. Blood pressure was measured using an Omron® automated blood pressure monitor following standard procedures. Three measures were taken and the mean of the second and third readings was considered for analysis.^20^ Diabetes was defined as self-reported diagnosis of diabetes, use of hypoglycemic agents in the last two weeks, fasting blood glucose pressure ≥7.0 mmol/L, plasma glucose of 11.1 mmol/L two hours after a standard oral glucose load, or glycated hemoglobin (HbA1c) level ≥ 6,5%. Blood glucose level was determined by the hexokinase method (ADVIA Chemistry; Siemens, Deerfield, Illinois, USA), and HbA1c was measured by liquid chromatography (Bio-Rad Laboratories, Hercules, California, USA). The use of antihypertensive and antidiabetic agents was assessed by patients' self-report, and information in drug prescriptions and drug labels. Total cholesterol and HDL cholesterol levels were measured using the ADVIA 1200 Siemens®. All laboratory parameters were determined in the same laboratory, after a median fasting time of 12 hours (10h – 14h).^21^

### Explanatory Variable

NC was measured using an inelastic tape (mm) above the cricothyroid cartilage, perpendicular to the long axis of the neck, with participant in sitting position. NC was used as continuous variable (cm) and categorized into quartiles.

### Covariables

The covariables included were sociodemographic data – age range (35-44, 45-54, 55-64, 65-74 years), used in the population description and as continuous variable in the regression models; self-reported race/skin color (white, *pardo*, black, yellow, indigenous) and educational attainment (undergraduate degree, high school, completed elementary school, some elementary school).

Health-related behaviors were also analyzed as covariables. Self-reported weekly use of alcohol was assessed considering number of doses and types of beverages and classified into ‘no consumption', ‘moderate' and ‘heavy drinking'. High consumption was defined as ≥ 210g for men and ≥ 140g for women, and any consumption below these values was considered moderate. Leisure-time physical activity was assessed using the long version of the International Physical Activity Questionnaire (IPAQ), and categorized by the sum of the time spent in each activity, weighed by the activity intensity (high: ≥ 3000 MET-min/week, moderate: 600-3000 MET-min/week, low: <600 MET-min/week).^22^

Body mass index (BMI) (kg/m^2^) and waist circumference (WC) (cm) were also assessed and described as categorical variables. BMI values were categorized into normal weight (BMI<25), overweight (BMI ≥25 and <30) and obesity (BMI≥30). WC was measured at the midpoint between the lowest rib and the iliac crest^24^ and classified as adequate or inadequate (≥88 cm for women and ≥102 cm for men).^23^ Both measures were used as continuous variables in the regression models. Measures were taken in fasting conditions using standard procedures.

### Statistical Analysis

Characteristics of the study population and components of the FGRS were described as absolute and relative frequencies (categorical variables), and mean and standard deviation (SD) (continuous variables with normal distribution) or median and interquartile range (continuous variables without normal distribution). Normality of data distribution was tested by the Shapiro-Wilk test. The Pearson's chi-square test was used for comparison of frequencies, the unpaired t-test used for comparison of means and the Mann-Whitney test used for comparison of medians. The one-way ANOVA test followed by the Bonferroni post-hoc test was performed to detect significant differences in NC values by 10-year cardiovascular risk (low risk <6%, intermediate ≥ 6% and ≤ 20% and high >20%). The level of significance adopted was 5%.

The magnitude of the association between NC and 10-year CVD risk was estimated using generalized linear models (GLM), which are a flexible generalization of ordinary linear regression models, that allow for non-normal errors and default link function.^25^ The GLM for the gamma distribution and logarithmic function was used. The results represent the arithmetic mean ratio (AMR), obtained by the exponentiation of the regression coefficients.

First, the gross association between NC (continuous) and the 10-year risk for CVD (model 0) was estimated. Then, the multivariate models were calculated, with successive adjustments for age, race/skin color, and educational attainment (Model 1), physical activity and alcohol consumption (model 2), BMI (model 3) and WC (model 4). In addition, in the estimation of the magnitude of the association of NC and the 10-year cardiovascular risk, NC was categorized in quartiles, and the multivariate analysis performed using the same sequence of adjustments. All analyses were stratified by sex.

Sensitivity analysis was performed by exclusion of those participants who were taking hypolipemic agents, corticoids, contraceptive pills, or women in hormone replacement therapy. The use of medications was assessed by self-report, drug prescriptions and drug labels on the day of the interview.

Statistical analysis was performed using the Stata software version 13 (Stata Corporation, College Station, USA).

## Results

Data of 13,920 participants were analyzed. Mean age was 51.7 years (SD ± 7.6), and 55% were women. Most participants reported a white race/skin color and completed higher education. The prevalence of overweight was higher in men, whereas the prevalence of obesity higher in women. An inadequate WC was more frequent in women than men (44.3% vs, 25.5%). Mean NC was 39.5 ± 3.6 cm in men and 34 ±2.9 cm in women (Table 1).

**Table 1:** Characteristics of the study population by sex, ELSA-Brasil, 2008-2010

Characteristics		Men		Women		p value
		(n=6,261)		(n=7,659)		
		n	%	n	%	
**Age (years)**						
	35-44	1,481	23.7	1,708	22.3	0.009*
	45-54	2,518	40.2	3,086	40.3	
	55-64	1,634	26.1	2,165	28.3	
	65-75	628	10.0	700	9.1	
**Self-reported race/skin color**						
	White	3,299	52.7	3,981	52	<0.001*
	*Pardo*	1,888	30.2	2,041	26.7	
	Black	870	13.9	1,351	17.6	
	Yellow	120	1.9	225	2.9	
	Indigenous	84	1.3	61	0.8	
**Educational attainment**						
	Completed higher school	3,162	50.5	4,245	55.4	<0.001*
	Completed high school	2,094	33.5	2,744	35.8	
	Completed elementary school	516	8.2	396	5.2	
	Some elementary school	489	7.8	274	3.6	
**Alcohol consumption**						
	Moderate	3,994	63.8	4,673	61.0	<0.001*
	None/former user	1,486	23.7	2,718	35.5	
	Heavy drinking	781	12.5	268	3.5	
**Leisure-time physical activity**						
	Mild	4,596	73.4	6,100	79.7	<0.001*
	Moderate	1086	17.4	1,143	14.9	
	High	579	9.2	416	5.4	
**Body mass index (BMI) (Kg/m**^2^)						
	Normal weight	2,179	34.8	3,040	39.7	<0.001*
	Overweight	2,819	45.0	2,756	36.0	
	Obesity	1,263	20.2	1,863	24.3	
**Waist circumference (cm)**						
	Adequate	4,662	74.5	4,270	55.7	<0.001*
	Inadequate	1,599	25.5	3,389	44.3	
**Neck circumference (cm), mean (±SD)**		39,5	(±3.6)	34,0	(±2.9)	<0.001†
**10-year cardiovascular risk score (%), median (1st/3rd quartile)**		11,3	(6.2- 19.9)	4,4	(2.4-8.3)	<0.001‡

BMI: normal weight < 24.9 kg/m²; overweight: 25.0 – 29.9 kg/m^2^; Obesity: ≥ 30 kg/m^2^.

Waist circumference: Inadequate: ≥ 88 cm (women) and ≥ 102 cm (men). Adequate < 88 cm (women) and < 102 cm (men).

* Pearson's chi-square test,

† unpaired t-test,

‡ Mann-Whitney test.

Results of the FGRS components are described in Table 2. Mean NC, grouped in risk categories (low risk <6%, intermediate ≥6% and ≤20%, and high risk >20%), increased with the increment of the 10-year CVD risk in both sexes (Figure 1).

**Table 2:** Components of the Framingham Global Risk Score in men and women, ELSA-Brasil, 2008-2010

Risk factors	Men (n=6.261)		Women (n=7.659)		p value
Age (years), mean (±SD)	51.7	±11.5	51.8	±10.0	0.53†
Total cholesterol (mg/dL), median (1^st^ and 3^rd^ quartiles)	210	(185-239)	214	(189-241)	<0.001‡
HDL cholesterol (mg/dL), median (1^st^ and 3^rd^ quartiles)	49	(43-57)	60	(51-70)	<0.001‡
Use of antihypertensive agents, n (%)	1.687	26.9	2.071	27.0	0.90*
Systemic arterial pressure, mean (±SD)	125.3	±20.9	117.2	±18.9	<0.001†
Diabetics, n (%)	1.200	19.2	1.068	14.0	<0.001*
Smokers, n (%)	889	14.4	927	12.1	<0.001*

* Pearson chi-square test,

† unpaired t-test,

‡ Mann-Whitney test.

**Figure 1:** Mean (cm) neck circumference by 10-year cardiovascular disease risk stratification in men and women, ELSA-Brasil, 2008-2010. Low risk: <6%; intermediate risk: ≥ 6% and ≤ 20%, high risk: > 20%. One-way ANOVA and Bonferroni post-hoc test.

Table 3 shows the results of the regression models with the variable NC. A one-centimeter increase in NC was associated with an increment of 5% (95% 1.04-1.05) in the mean 10-year CVD risk (Model 0). This association remained statistically significant after all adjustments (AMR: 1.03; 95%CI:1.02-1.03) (Model 4). Among women, the increase of one centimeter in NC was associated with an increment of 11% in the mean 10-year CVD risk (95%CI: 1.10-1.12) (Model 0). After all adjustments (Model 4), the one-centimeter increase in NC was associated with an increment of 5% (95%CI: 1.04 – 1.06) in the mean 10-year CVD risk (Table 3).

**Table 3:** Multivariate analysis of the association between neck circumference and the risk of developing cardiovascular disease in 10 years in men and women, ELSA-Brasil, 2008-2010

	Men	Women
	AMR (95%CI)	AMR (95%CI)
Model 0	1.05 (1.04 – 1.05)	1.11 (1.10 – 1.12)
Model 1	1.06 (1.05 – 1.06)	1.09 (1.08 – 1.10)
Model 2	1.06 (1.05 – 1.06)	1.09 (1.08 – 1.10)
Model 3	1.03 (1.02 – 1.04)	1.07 (1.06 – 1.08)
Model 4	1.03 (1.02 – 1.03)	1.05 (1.04– 1.06)

AMR (95%CI): arithmetic mean ratio obtained by generalized linear models and respective 95% confidence intervals. Model 0: unadjusted arithmetic mean ratio; Model 1: Adjusted for age, self-reported race/skin color and schooling; Model 2: Model 1 + adjusted for alcohol consumption and leisure-time physical activity; Model 3: Model 2 + adjusted for body mass index; Model 4: Model 3 + waist circumference.

Results of the regression models using the NC grouped into quartiles are presented in Table 4. In all models, there was a gradual increase in the arithmetic mean of the 10-year CVD risk from the first to the fourth quartile, achieving an increase of 18% in those located in the last quartile (95%CI: 1.13-1.24) among men and of 35% (95%CI: 1.28-1.43) among women (Model 4).

**Table 4:** Multivariate analysis of the association between neck circumference grouped into quartiles and the 10-year cardiovascular disease risk in men and women, ELSA-Brasil, 2008-2010

	Men AMR (95%CI)				Women AMR (95%CI)			
	Q1	Q2	Q3	Q4	Q1	Q2	Q3	Q4
Model 0	1.00	1.14(1.08-1.21)	1.21(1.14-1.28)	1.42(1.34-1.51)	1.00	1.23(1.16-1.31)	1.59(1.49-1.70)	2.04(1.91-2.18)
Model 1	1.00	1.14(1.10-1.19)	1.30(1.25-1.35)	1.52(1.47-1.58)	1.00	1.16(1.11-1.21)	1.37(1.31-1.43)	1.78(1.71-1.86)
Model 2	1.00	1.14(1.10-1.18)	1.28(1.24-1.33)	1.49(1.43-1.54)	1.00	1.16(1.11-1.20)	1.36(1.30-1.41)	1.76(1.69-1.84)
Model 3	1.00	1.07(1.04-1.11)	1.16(1.11-1.21)	1.24(1.18-1.30)	1.00	1.10 (1.06-1.15)	1.25(1.19-1.31)	1.50(1.42-1.58)
Model 4	1.00	1.05(1.02-1.09)	1.12(1.08-1.17)	1.18(1.13-1.24)	1.00	1.06(1.02-1.11)	1.17(1.12-1.23)	1.35(1.28-1.43)

AMR (95%CI): arithmetic mean ratio obtained by generalized linear models and respective 95% confidence intervals Q1, Q2, Q3, Q4: Interquartile range. Model 0: unadjusted arithmetic mean ratio; Model 1: Adjusted for age, self-reported race/skin color and schooling; Model 2: Model 1 + adjusted for alcohol consumption and leisure-time physical activity; Model 3: Model 2 + adjusted for body mass index; Model 4: Model 3 + waist circumference.

Analyses of sensitivity showed that the exclusion of participants using hypolipemic agents, corticoids, and of women taking contraceptive agents or in hormone replacement therapy did not change the results.

## Discussion

The findings of the present study indicate a direct association between NC and 10-year CVD risk, independently of others potential cofounding factors and body adiposity measures, particularly BMI and WC, in individuals free of CVD. This was corroborated by the results of the analysis of NC grouped into quartiles, which indicated a dose-response gradient. The magnitude of the associations between NC (both continuous and in quartiles) and the 10-year CVD risk was higher in women than men.

Our results pointed to a direct association between NC and the 10-year CVD risk. We found only one study that investigated the relationship between NC and the risk for CVD in 10 years estimated by the Framingham coronary heart disease risk score. This study, that included only 100 individuals free of CVD, pointed out a positive correlation between NC and the 10-year CVD risk.^14^ However, previous studies have indicated a positive independent association between NC and intima-media thickening (IMT),^26^ a marker of subclinical atherosclerosis, predictive of cardiovascular risk. The baseline analysis of the ELSA-Brasil study also showed an association between NC and IMT, but did not find an association between NC and coronary artery calcification, another measure of subclinical atherosclerosis.^27^^,^^28^

The mechanisms of how neck adipose tissue can contribute to the occurrence of cardiovascular outcomes are not well established.^29^ Neck adipose tissue is considered an ectopic fat depot,^1^ which may explain part of the systemic effect. The formation of ectopic fat depots in several organs, including subcutaneous fat in neck, result from deposition of triglycerides in non-adipose tissue cells that normally contain small amounts of fat and seem relevant for cardiovascular risk,^30^^,^^31^ especially ectopic fat depositions in the pericardium and liver.^32^ Dysfunctional activity of ectopic fat is associated with oxidative stress, endothelial dysfunction, increased secretion of pro-inflammatory cytokines and reduced release of the anti-inflammatory adiponectin, leading to chronic inflammation and altered lipid metabolism^33^ involved in the atherosclerotic process. Evidence has supported the association of greater NC and inflammatory markers, notably plasma proteins of the complement pathway, (C3 and C4), C-reactive protein, interleukin-6 and tumor necrosis factor alpha (TNF-α),^34^ and markers of endothelial dysfunction such as E-selectin.^9^ Also, ectopic fat seems to be a key element that differs metabolically healthy from metabolically non-healthy obese subjects.^26^

In addition, most of blood vessels, including carotid arteries, are involved by the perivascular adipose tissue, which helps in the vascular tone and endothelial function regulation.^35^ As this tissue increases and becomes dysfunctional, there seems to be a direct pro-inflammatory effect on the carotid arteries, which may explain the increased cardiovascular risk related to the increment in NC.30^30^ It is worth pointing out that NC is a renowned risk factor for sleep obstructive apnea, which, in turn, is associated with increased risk for CVD and type 2 diabetes.^36^

Our findings revealed stronger associations between NC and 10-year CVD risk in women compared with men. In agreement with this, results of the Framingham Heart Study showed a strong association between elevated NC and dyslipidemia and hypertension among women,^11^ and an increased risk of developing diabetes associated with increased NC was reported in women.^37^ On the other hand, NC was more strongly associated with the risk for coronary artery disease in 10 years, estimated by the Framingham Coronary Artery Disease Risk Score in men than women.^14^ A similar association between NC and cardiometabolic changes between men and women has also been reported.^10^

It is possible that differents patterns of neck fat accumulation,^38^ subcutaneous fat distribution,^39^ and metabolism of free fatty acids^11^ explain the differences between genders in the present study and in others. In addition, while women are more likely to develop subcutaneous fat, Men have a higher tendency to accumulate abdominal visceral fat.^40^ Upper-body subcutaneous fat delivers more free acids to the systemic circulation as compared with visceral fat,^41^ and increased levels of free fatty acids in plasma contribute to higher insulin resistance, increased secretion of very low density lipoprotein and triglycerides, and induction of oxidative stress and blood pressure elevation.^11^^,^^41^ It is also known that neck adipose tissue is distributed into three different compartments – posterior, subcutaneous, and perivertebral compartments – that contribute differently to metabolic risk.^38^ While women tend to accumulate subcutaneous neck fat, greater neck fat accumulation in the other compartments has been observed in men.^30^ Posterior and subcutaneous neck adipose tissue seem to be more associated with metabolic syndrome in women.^38^

In the present study, calculation of the 10-year CVD risk showed a lower median score in women than men, which may be explained by higher HDL cholesterol levels, lower systolic blood pressure, and lower prevalence of diabetes and smoking habits. This result corroborates the differences between gender in the exposure to cardiovascular disease risk factors,^30^^,^^38^^,^^40^^,^^41^ and greater likelihood of women to seek out health care than men.^42^

Results of the present study indicate that increased NC may contribute to the prediction of the 10-year CVD risk, independently of BMI and WC, which are the most studied measures of adiposity. Some authors have suggested advantages of NC over WC, since the former is simple, easy-to-perform and less likely to measuring errors.^43^ It is interesting to note that our findings suggest that NC may be more strongly associated with 10-year CVD risk, since after all adjustments, the increase of 1 cm in NC was associated with a greater increment in the arithmetic mean of CVD risk as compared with the increase of 1cm in WC [women: 5% (AMR:1.05; 95%CI:1.04-1.06) versus 3% (AMR:1.03; 95%CI:1.01-1.02) and men: 3% (AMR:1.03; 95CI%:1.02-1.03) versus 1% (AMR: 1.01; 95%CI:1.01-1.02), respectively]. This is reinforced by a recent meta-analysis showing an association of NC with coronary artery disease.^44^

Strengths of our study include the sample size, the methodological rigor, adjustment for potential cofounding factors, and the investigation of the association between NC and 10-year CVD risk, measured by the FGRS, which is a known cardiovascular risk predictor already used in clinical practice and, similar to NC, does not include invasive measurements.

Limitations of the study include the cross-sectional nature of the analysis, the lack of validation of the FGRS for the Brazilian population, and the fact that NC was measured only once. The presence of residual confounding cannot be excluded.

## Conclusion

Increased NC was positively associated with the 10-year CVD risk, regardless of measures of total and visceral adiposity. These findings suggest that NC may contribute to the prediction of cardiovascular risk, over and above traditional anthropometric parameters, such as BMI and WC. Longitudinal analysis will contribute to clarify the causal role of NC in the cardiovascular risk.
